# A phase I study of the PD-L1 inhibitor, durvalumab, in combination with a PARP inhibitor, olaparib, and a VEGFR1–3 inhibitor, cediranib, in recurrent women’s cancers with biomarker analyses

**DOI:** 10.1186/s40425-019-0680-3

**Published:** 2019-07-25

**Authors:** Alexandra S. Zimmer, Erin Nichols, Ashley Cimino-Mathews, Cody Peer, Liang Cao, Min-Jung Lee, Elise C. Kohn, Christina M. Annunziata, Stanley Lipkowitz, Jane B. Trepel, Rajni Sharma, Lekha Mikkilineni, Margaret Gatti-Mays, William D. Figg, Nicole D. Houston, Jung-Min Lee

**Affiliations:** 1 Women’s Malignancies Branch, Center for Cancer Research, National Cancer Institute, Bethesda, MD USA; 2 Johns Hopkins Hospital Department of Pathology, Baltimore, MD USA; 3 Johns Hopkins Hospital Department of Oncology, Baltimore, MD USA; 4 Genitourinary Malignancies Branch, National Cancer Institute, Bethesda, MD USA; 5 Genetics Branch, National Cancer Institute, Bethesda, MD USA; 6 Developmental Therapeutics Branch, Center for Cancer Research, National Cancer Institute, Bethesda, MD USA; 7 Clinical Monitoring Research Program Directorate, Frederick National Laboratory for Cancer Research sponsored by the National Cancer Institute, Bethesda, MD USA

**Keywords:** Ovarian cancer, Immune checkpoint inhibitor, PARP inhibitor, VEGF inhibition

## Abstract

**Background:**

Strategies to improve activity of immune checkpoint inhibitors are needed. We hypothesized enhanced DNA damage by olaparib, a PARP inhibitor, and reduced VEGF signaling by cediranib, a VEGFR1–3 inhibitor, would complement anti-tumor activity of durvalumab, a PD-L1 inhibitor, and the 3-drug combination would be tolerable.

**Methods:**

This phase 1 study tested the 3-drug combination in a 3 + 3 dose escalation. Cediranib was taken intermittently (5 days on/2 days off) at 15 or 20 mg (dose levels 1 and 2, respectively) with durvalumab 1500 mg IV every 4 weeks, and olaparib tablets 300 mg twice daily. The primary end point was the recommended phase 2 dose (RP2D). Response rate, pharmacokinetic (PK), and correlative analyses were secondary endpoints.

**Results:**

Nine patients (7 ovarian/1 endometrial/1 triple negative breast cancers, median 3 prior therapies [2–6]) were treated. Grade 3/4 adverse events include hypertension (1/9), anemia (1/9) and lymphopenia (3/9). No patients experienced dose limiting toxicities. The RP2D is cediranib, 20 mg (5 days on/2 days off) with full doses of durvalumab and olaparib. Four patients had partial responses (44%) and 3 had stable disease lasting ≥6 months, yielding a 67% clinical benefit rate. No significant effects on olaparib or cediranib PK parameters from the presence of durvalumab, or the co-administration of cediranib or olaparib were identified. Tumoral PD-L1 expression correlated with clinical benefit but cytokines and peripheral immune subsets did not.

**Conclusions:**

The RP2D is tolerable and has preliminary activity in recurrent women’s cancers. A phase 2 expansion study is now enrolling for recurrent ovarian cancer patients.

**Trial registration:**

ClinicalTrials.gov identifier: NCT02484404. Registered June 29, 2015.

**Electronic supplementary material:**

The online version of this article (10.1186/s40425-019-0680-3) contains supplementary material, which is available to authorized users.

## Introduction

Immunotherapy has emerged as a major therapeutic modality in oncology. However, the majority of patients with women’s cancers do not derive benefit from immune checkpoint blockade monotherapy, creating the need to optimize combination treatment strategies [1].

Data suggest increased DNA damage by radiation or DNA repair inhibitors promotes local antigen release resulting in systemic anti-tumor immune responses [2]. Such neoantigen release and high tumor mutational burden (TMB) are shown to be associated with clinical response to immune checkpoint inhibition in solid tumors [3].

PARP inhibitors (PARPi) are one of the active new drug families in the drug armamentarium for women’s cancers and may modulate immune responses. PARPi, such as olaparib and talazoparib were shown to induce PD-L1 expression in breast cancer in vitro and in vivo models [4, 5]. Also, increased DNA damages from PARPi exposure may yield greater TMB, potentially increasing neoantigens, and affect the immune milieu, complementing the clinical benefit of immune checkpoint blockade in subsets of recurrent women’s cancer.

Angiogenesis pathways interact with both DNA repair mechanisms and immune activity. Tumor hypoxia induces downregulation of genes involved in DNA repair, e.g.*,* RAD51 and BRCA1, leading to further DNA damages, genomic instability, and cell death [6]. VEGF suppresses lymphocyte trafficking across endothelia into tumor deposits and sites of inflammation to promote vessel growth [7]. Combining inhibition of DNA repair and angiogenesis pathways therefore may modulate the immune response by increasing DNA damage and TMB and by attenuating immunosuppressive microenvironments.

The combination of olaparib and cediranib, a VEGFR1–3 inhibitor, has been demonstrated to be clinically superior to olaparib monotherapy in recurrent platinum-sensitive ovarian cancer [8]. We extended this concept with our hypothesis that reduced VEGF signaling by cediranib and increased DNA damages by olaparib would complement anti-tumor activity of the immune checkpoint inhibitor. Durvalumab (MEDI4736) is a selective, high-affinity human IgG1 monoclonal antibody that blocks PD-L1 binding to PD-1 and CD80, thereby enhancing the function of tumor-directed T cells [9]. We previously reported the safety data and recommended phase 2 doses (RP2D) of the doublets of durvalumab in combination with olaparib or intermittent cediranib in recurrent women’s cancers [10]. We now report safety, RP2D and pharmacokinetics (PK)/pharmacodynamics (PD) findings of the 3-pathway modulation using durvalumab in combination with olaparib and cediranib.

## Patients and methods

### Study design and patients

The trial was approved by the institutional review board of the Center for Cancer Research, National Cancer Institute (ClinicalTrials.gov identifier: NCT02484404). Patients eligible for the study had histologically confirmed advanced breast or gynecologic malignancies, measurable by Response Evaluation Criteria in Solid Tumors (RECIST) v1.1. Prior exposure to PARPi or angiogenesis inhibition was eligible but previous treatment with immune checkpoint blockade was not allowed. Patients had to have controlled hypertension with no more than three antihypertensives, and good end-organ function. Germline *BRCA* mutation status was requested at baseline. All patients provided written informed consent before enrollment.

Eligible patients received all three drugs in a 3 + 3 dose-escalation format as outlined in Table 1. Patients safety was assessed in an ongoing fashion using the Common Terminology Criteria for Adverse Events v4. Response was assessed every two cycles by imaging using RECIST v1.1 criteria. Study treatment was discontinued for progression of disease, intercurrent illness, adverse events (AEs) not recovering to ≤ grade 1 within 14 days, or patient withdrawal of consent.

**Table 1:** Dose levels

Dose level (DL)	Durvalumab (IV)	Olaparib tablet (oral)	Cediranib (oral)
DL − 1	1500 mg every 4 weeks	200 mg twice daily	15 mg once daily (5 days on/2 days off)
DL 1 (starting dose)	1500 mg every 4 weeks	300 mg twice daily	15 mg once daily (5 days on/2 days off)
DL 2	1500 mg every 4 weeks	300 mg twice daily	20 mg once daily (5 days on/2 days off)

### Definitions of dose-limiting toxicity and maximum tolerated dose

The primary end point was to determine RP2D of the 3-drug combination, defined by the maximum tolerated dose (MTD) or the highest protocol-defined dose in the absence of dose-limiting toxicity (DLT). DLT was defined as grade 3 or 4 non-hematologic and grade 4 hematologic AEs related to study medications occurring during the first cycle (28 days). The MTD was defined as the highest dose level at which one or fewer of six patients experienced a DLT. If the observed AE could be specifically attributed to only one of the drugs, that drug was held while the patient continued to receive the drug not associated with the observed AE. Treatment-related serious AEs occurring within 90 days after the last dose of study drugs were reported.

Patients were asked to take blood pressure (BP) twice-daily and given anti-hypertensive medication(s) if two consecutive readings > 140/90 mmHg. Diarrhea episodes were also recorded in a diary and treated with loperamide per protocol unless associated with immune-mediated colitis.

### Pharmacokinetic studies

Plasma samples were collected at 0.5, 1, 2, 4, 8, and 12 h after the first dose(s) of olaparib and cediranib and immediately prior to the second dose of olaparib (approximately 12 h after the first daily dose), on cycle 1 day 1 before durvalumab administration, and again after durvalumab on cycle 2 day 1. Plasma was separated and stored at -80 °C until measurement. The lower limit of quantitation of the assay is 0.5 ng/mL for both olaparib and cediranib using previously reported validated methods [10, 11]. A noncompartmental approach to calculating pharmacokinetics data was employed using Phoenix® WinNonlin v6.4 (Certara Pharsight, Cary, NC).

### Archival tissue PD-L1 expression and tumor infiltrating lymphocytes evaluation

PD-L1 expression on archival tissue was a prespecified exploratory end point. PD-L1 labeling of cancer cells and tumor-infiltrating lymphocytes (TILs) was evaluated in archival tissue samples by immunohistochemistry (IHC). The degree of TIL was assessed as a percentage of stromal and intratumoral space occupied by TIL. Unstained slides of the primary tumor sample from all 9 patients were labeled for PD-L1 by IHC with antibody SP263 (monoclonal rabbit; Ventana Medical Systems Inc., Tucson, AZ) on an automated platform [10]. PD-L1 positivity was defined as labeling of ≥1% carcinoma cells or TIL. The PD-L1 labeling by the carcinoma cells and the TIL was assessed and recorded separately.

### Pharmacodynamic studies

#### Immune subsets and functional markers analysis

Peripheral blood mononuclear cells (PBMCs) were collected at baseline (cycle 1 day 1) and on therapy (cycle 1 day 15 and cycle 3 day 1). PBMCs were processed within 2 h and assessed for immune subsets and functional markers using multiparameter flow cytometry as described [12]. CD8+ T cells, CD4+ T cells, Tregs, monocyte subsets and MDSCs were studied. Expression of CTLA-4, T cell immunoglobulin and mucin-domain containing-3 (TIM-3) and PD-1 on CD8+ T cells and Tregs, CD40+ MDSCs, and monocytes HLA-DR and PD-L1 were evaluated as described previously [13]. All analyses were performed using multiparametric flow cytometry (MACSQuant; Miltenyi Biotec, Bergisch Gladbach, Germany), and data were analyzed using FlowJo software ​v.10.0.7 (FlowJo LLC, Ashland, OR). Flow cytometric data were quantified either as a percentage of a defined cell population, or the median fluorescence intensity (MFI).

#### Cytokine studies

Plasma samples collected pre- and on treatment were analyzed by ELISA [14]. The pro-inflammatory cytokines IFN γ, IL 10, IL 12, IL 2, IL 6, IL 8 and TNFα were examined.

### Statistical analyses

All statistical tests used two-sided significance level 0.05, adjusted for multiple comparisons, using GraphPad Prism software v6.0 (GraphPad Software Inc., La Jolla, CA). Paired *t*-tests were performed in a parametric manner.

## Results

### Patient characteristics

Nine patients were enrolled between June 16, 2016 and January 3, 2017. The majority of the patients had ovarian carcinoma (6/9 [66%]) and all but one had germline *BRCA* wild-type recurrent platinum-resistant disease. Baseline patient characteristics are listed in Table 2.

**Table 2:** Baseline characteristics

Characteristics	*N* = 9
Age, median (range)	59 year-old (44–73)
BRCA mutation status (germline mutation/wild type/unknown)	1/7/1
Tumor type	
OvCa (HGSOC/Clear cell/Mixed Mullerian/ Mixed Serous and Endometrioid)	6 (2/2/1/1)
Platinum sensitivity in OvCa (sensitive/resistant)	2/5
Primary Peritoneal cancer (platinum-resistant)	1
Endometrial carcinoma (MSI low)	1
Triple Negative Breast Cancer	1
ECOG Performance Status (0/1/2)	3/6/0
Number of previous treatments, median (range)	2 (2–6)
Prior PARPi (n)	0
Prior bevacizumab (n)	2

*Abbreviations*: *N* number, *OvCa* ovarian cancer, *HGSOC* high-grade serous OvCa, *MSI* microsatellite instability, *PARPi* poly ADP ribose polymerase inhibitor

### Dose optimization and toxicities

The dose level 2 was identified as the RP2D, with durvalumab 1500 mg IV every 4 weeks in combination with olaparib 300 mg twice daily and cediranib 20 mg daily (5 days on/2 days off). No DLT was observed during the treatment with a 3-drug combination. One patient required a one dose level reduction of olaparib during cycle 5, due to recurrent grade 3 anemia. The most common AEs related to treatment were hematologic and gastrointestinal toxicities. All patients had at least one any-grade AE and those are summarized in Table 3.

**Table 3:** Treatment related adverse events by maximum grade per patient

Adverse event	Grade 1	Grade 2	Grade 3	Grade 4
Hematological				
Lymphopenia	1	2	3	0
Anemia	2	1	2	0
Thrombocytopenia	2	0	0	0
Neutropenia	1	1	0	0
Gastrointestinal				
Anorexia	2	0	1	0
Nausea	5	0	0	0
Vomit	3	0	0	0
Diarrhea	5	0	0	0
GERD	0	1	0	0
Dyspepsia	1	1	0	0
Endocrinology and Chemistry				
Increased creatinine	1	2	1	0
Hypothyroidism	1	1	0	0
Proteinuria	1	0	0	0
Increase ALT/AST	4	0	0	0
Increase Alkaline Phosphatase	2	0	0	0
Cardiovascular				
Hypertension	0	3	1	0
Syncope	0	0	1^a^	0
DVT	0	1^b^	0	0
Other				
Fatigue	6	2	0	0
Dyspnea	1	0	0	0
Headache	1	0	0	0
Arthralgia	2	0	0	0
Dizziness	2	0	0	0
Gastric hemorrhage	0	1	0	0
Hoarseness	1	0	0	0

Anemia occurred in 5 of 9 patients, one with grade 3 anemia required olaparib dose reduction. One patient was taken off study treatment for extensive progression of disease after 3 cycles of treatment and developed multifactorial causes for renal failure, grade 3 creatinine elevation and grade 3 anemia at the time. This patient also developed a new-onset DVT in lower extremity after cycle 3 of treatment, thus cediranib was discontinued but other two drugs were continued

*Abbreviations*: *GERD* gastroesophageal reflux disease, *AST* aspartate aminotransferase, *ALT* alanine aminotransferase, *DVT* deep venous thrombosis

^a^Unlikely related to study drugs – determined to be a non-drug related vasovagal episode after extensive cardiovascular investigation including brain imaging

^b^Possibly related to cediranib and disease, cediranib was discontinued after grade 2 DVT event but durvalumab and olaparib were continued

### Clinical activity

**Fig. 1:**
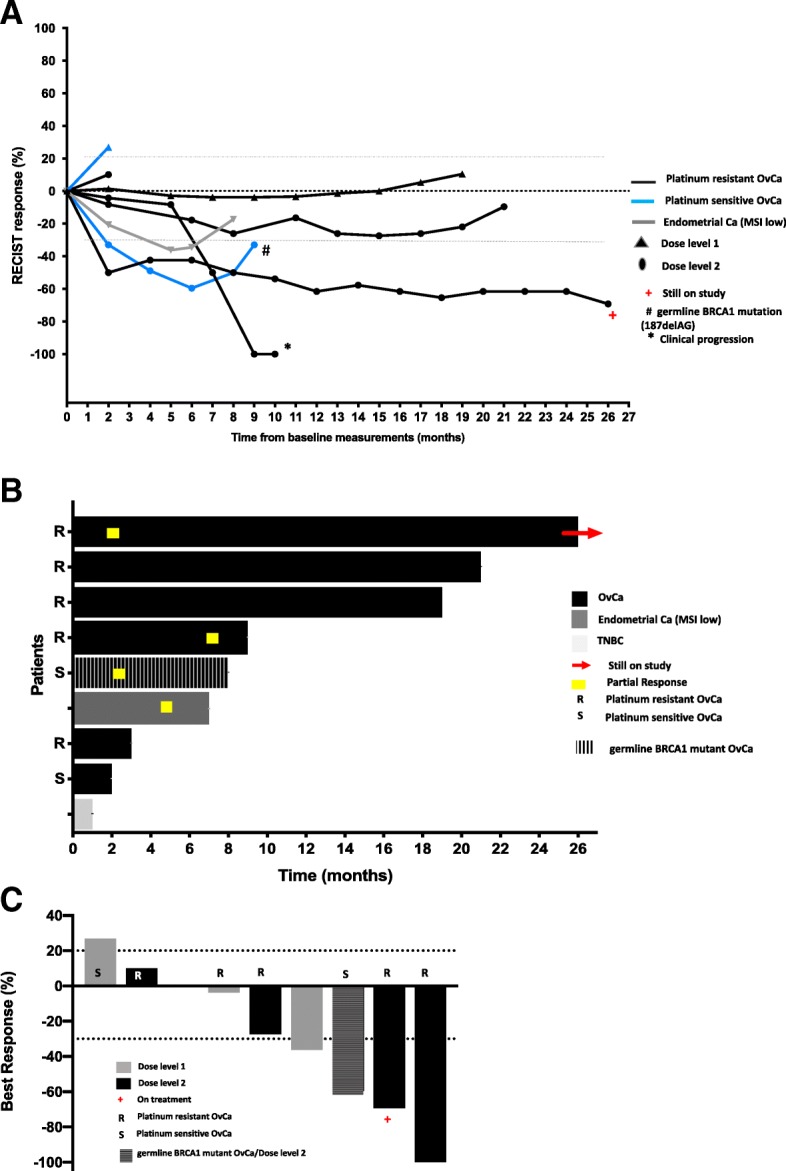
Changes in tumor size and duration on the treatment. **a** Changes in tumor size on the study treatment. **b** Duration in the study. **c** Best response. One patient with TNBC had clinical progressive disease before first response evaluation imaging and is represented as zero (0) in **a** and **b**. Abbreviations: S: platinum-sensitive recurrent ovarian cancer, R: platinum-resistant recurrent ovarian cancer. OvCa: ovarian cancer. TNBC: triple negative breast cancer

The objective response rate was 44% (4/9) with all partial responses (PRs), lasting a median of 8.5 months [range 7 to 26 months]. One patient with PR was still receiving the study treatment at the time of data cutoff (October 25, 2018), with 26+ months of continuous treatment. Three patients (33%) had stable disease (SD), 2 of them lasting 19 and 21 months, respectively. The clinical benefit rate, defined herein as complete response [CR] + PR + SD ≥6 months, was 67% (6/9). Changes from baseline in tumor size and duration of response are shown in Fig. 1.

### PK studies

No clinically significant effects on olaparib PK from the presence of durvalumab, or the co-administration of cediranib were identified. Olaparib exposures were comparable before and after durvalumab, either alone or with cediranib, and clearance and volume of distribution were unchanged. Similarly, with the intermittent dosing schedule for cediranib (5 days on/2 days off), no statistically significant changes in cediranib PK parameters were caused by the presence of durvalumab, olaparib, and durvalumab plus olaparib (Additional file 1: Figure S1).

### Archival tissue TIL infiltration and PD-L1 expression

Some degrees of TIL infiltration were present in all tumors: focal (< 5% tumoral TIL; 3/9), moderate (5–50% tumoral TIL; 5/9) and brisk TIL infiltration (≥50% tumoral TIL; 1/9). The majority of tumors (7/9) had PD-L1 positive carcinoma cells, and most cases (8/9) contained PD-L1 positive TIL. All seven patients with PD-L1 positive carcinoma cells had SD or PR as best response, whereas two patients with PD-L1 negative carcinoma cells had PD (*p* = 0.03) (Additional file 2: Figure S2 and Additional file 5: Table S1). The two tumors with no carcinoma cell PD-L1 labeling, contained PD-L1 positive TIL. However, there was no statistically significant association between the degree of TIL infiltrate, the degree of TIL PD-L1 labeling, or the degree of carcinoma cell PD-L1 labeling and the duration of response.

### PD studies

#### Immune subsets and functional markers analysis

PBMCs of all patients (*n* = 9) were evaluated for immune subsets and functional markers. A transient increase in PD-L1 expression on total CD14+ monocytes was observed on cycle 1 day 15 (median MFI 1.8 [baseline] vs 3.0 [cycle 1 day 15]; *p* < 0.0001) which was not sustained on cycle 3 day 1 (Additional file 3: Figure S3B). Other innate and adaptive immune cells and functional markers were analyzed and did not demonstrate significant changes after treatment.

#### Cytokines

Changes in circulating proinflammatory cytokines (IFNγ, TNFα, IL2, IL6, IL8, IL10, and IL12) before and after treatment were evaluated. The median values of circulating cytokines were not statistically different between baseline and cycle 1 day 15 or cycle 3 day 1 (Additional file 4: Figure S4).

## Discussion

This is the first reported 3-drug combination therapy of immune checkpoint blockade with a PARPi and a VEGFR inhibitor. This study established the RP2D of 3-drug combination and demonstrates preliminary evidence of activity in heavily pretreated recurrent gynecologic cancer patients, particularly in ovarian cancer. We previously reported durvalumab with an intermittent cediranib schedule had greater tolerability without attenuating clinical benefit observed in the daily schedule [10]. We now observe a full dose olaparib can be added to that combination in a safe manner using prospective toxicity mitigation approaches. Unlike our prior observations where durvalumab affected Cmax and clearance of daily cediranib [10], PK parameters of cediranib and olaparib were not affected by the presence of durvalumab in this 3-drug combination.

The current study presents durable long-term activity and safety outcome of the 3-pathway modulation with a median of 8 months follow-up (range 1 to 26+ months) and a RR worthy of further investigation. Single agent olaparib has limited activity (4%) in heavily pretreated *BRCA* wild-type patients with platinum-resistant ovarian cancer [15]. Cediranib monotherapy also demonstrated a RR of 17% and a median PFS of 5.2 months in patients with platinum-resistant ovarian cancer, treated in the 2nd or 3rd line setting [16]. However, our data should be interpreted with a caution due to its small sample size and phase 1 single-arm dose escalation design, thereby preventing direct comparison of the combination with either drug alone.

There are limited published data on anti-tumor activity and safety of PD-1/PD-L1 blockade combinations with a PARPi or VEGF/VEGFR inhibition in women’s cancer. Preliminary results from two PARPi and PD-1/PD-L1 blockade combination trials were presented showing early clinical activity in subsets of recurrent ovarian cancer; 25% RR of niraparib and pembrolizumab in platinum-resistant ovarian cancer [17] and 72% RR of durvalumab and olaparib in germline *BRCA* mutant platinum-sensitive ovarian cancer patients [18]. Our group reported, at the 2018 European Society for Medical Oncology (ESMO) meeting, a 14% RR and 37% clinical benefit rate (as defined in Methods) of the combination durvalumab and olaparib, both in full doses, in heavily pretreated ovarian cancer patients, predominantly composed of *BRCA* wild-type and platinum-resistant disease [19]. A RR of 25% with niraparib and pembrolizumab was shown in women with platinum-resistant ovarian cancer [17], and in germline *BRCA* mutant platinum-sensitive ovarian cancer, a 72% RR with durvalumab and olaparib [18]. The single arm phase II study of bevacizumab and nivolumab in recurrent ovarian cancer presented at ESMO 2018 reported 16.7% RR and 55.3% CBR (defined by CR + PR + SD ≥ 6 months), with a median PFS of 5.3 months in platinum-resistant patients (18 patients) and 40% RR and 75% CBR, with a median PFS of 9.4 months in those with platinum-sensitive disease (20 patients), respectively [20]. These findings suggest a modest benefit of addition of either PARPi or bevacizumab to PD-1/PD-L1 blockade in germline *BRCA* wild-type ovarian cancer patients, warranting further investigation of our 3-drug combination strategy. Separately, phase 3 randomized trials of 3-pathway modulation (immune checkpoint inhibitor in combination with PARPi and VEGF inhibition) are now being investigated in the front-line setting for ovarian cancer treatment (e.g.*,* avelumab (PD-L1 inhibitor) + talazoparib (PARPi) + bevacizumab, ClinicalTrials.gov identifier: NCT03642132).

Defining predictive biomarkers of immune checkpoint inhibitor-based combination therapy is a major challenge. Our study suggests that presence of PD-L1 positive carcinoma cells in archival tissues may be associated with clinical benefit. In contrast, peripheral immune subsets analysis showed a transient increase of PD-L1 expression on total CD14+ monocytes after 2 weeks treatment of olaparib and cediranib although it did not last after 2 months treatment. An association of tumoral PD-L1 expression and response to immune checkpoint inhibitor therapy has been reported in ovarian carcinoma [21] and triple negative breast cancer (TNBC) [22]. The correlation of clinical benefit and expression of PD-L1 using archival tissues needs further validation, particularly in recurrent women’s cancers given preanalytical variabilities (e.g.*,* different IHC techniques, score systems, different antibodies [23, 24]) and possible dynamic changes of PD-L1 expression affected by prior therapies [25]. The present study is now being expanded to a phase 2 study for patients with recurrent ovarian cancer. We will prospectively examine the association of PD-L1 induction and TMB by PARPi with clinical response to immune checkpoint blockade using fresh core biopsies collected before and on-treatment.

In summary, our study demonstrates that the 3-drug combination is tolerable and active in heavily pretreated recurrent gynecologic cancer patients without germline *BRCA* mutation. This response may be associated with carcinoma cell PD-L1 labeling, but this requires further validation. The preliminary activity findings warrant further investigation and a single-arm phase II expansion study is now open to accrual for recurrent ovarian cancer patients (ClinicalTrials.gov identifier: NCT02484404).

## Additional files

Additional file 1: **Figure S1.** Pharmacokinetics effects of durvalumab on olaparib and cediranib. (A-B) Durvalumab did not affect olaparib PK Cmax or AUC. (C-D) The presence of durvalumab did not significantly affect cediranib PK. One patient’s PK data is missing due to no sample collection. One patient (red dot) showed abnormally low plasma concentrations that led to higher than normal CLss/F, possibly due to food effect on absorption. Abbreviations: AUC_INF_: area under the plasma concentration v. time curve from time zero to infinity. AUC/D: Area under the plasma concentration v. time curve normalized to dose. AUC_TAU_: AUC for the dosing interval for steady-state kinetics after durvalumab; 12 h for olaparib, 24 h for cediranib (PPTX 161 kb)

Additional file 2: **Figure S2.** Tumor infiltrating lymphocytes (TIL) and PD-L1 analysis by immunohistochemistry. (A-B) Patient B04 had a PR of 9 months duration; her primary HGSOC (arrow) showed PD-L1 positivity in the carcinoma cells, as well as within the TIL (star) (× 200). (C-D) Patient B09 experienced PD; her primary TNBC (arrow) did not display any PD-L1 labeling, and there were minimal TIL (< 5%) within the tumor bed. Abbreviations: PR: partial response, HGSOC: high grade serous ovarian carcinoma, TIL: tumor infiltrating lymphocytes, TNBC: triple negative breast cancer (PPTX 9168 kb)

Additional file 3: **Figure S3.** Peripheral immune subsets and functional markers. (A) CD8/CD4 ratio. (B) PD-L1 expression on total C14+ monocytes. Open dots: germinative BRCA mutated cases. Abbreviations: MFI: median fluorescence intensity. (PPTX 95 kb)

Additional file 4: **Figure S4.** Proinflammatory cytokines analysis. Plasma levels of pro-inflammatory cytokines (IFN γ, TNFα, IL 2, IL 6, IL 8 IL 10, and IL 12) were not changed significantly by the treatment. (PPTX 189 kb)

Additional file 5: **Table S1.** Pathologic characteristics and immune correlates. (DOCX 15 kb)

## Data Availability

All data generated or analysed during this study are included in this published article (and its additional files).

## Abbreviations

AE: Adverse event
CBR: Clinical benefit rate
CR: Complete response
DLT: Dose-limiting toxicity
IHC: Immunohistochemistry
MDSCs: Myeloid-derived suppressor cells
MFI: Median fluorescence intensity
MMR: Mismatch repair
MSI: Microsatellite instability
MTD: Maximum tolerated dose
PARP-inhibitor: Poly ADP ribose polymerase inhibitor
PBMCs: Peripheral blood mononuclear cells
PK: Pharmacokinetic
PR: Partial response
RP2D: Recommended phase 2 dose
RR: Response rate
SD: Stable disease
TIL: Tumor infiltrating lymphocytes
TMB: Tumor mutational burden
TNBC: Triple-negative breast cancer
Treg: Regulatory T cells
VEGFR 1–3 inhibitor: Vascular endothelial growth factor receptors 1–3 inhibitor
