# Supplementary material for: A phase I study of the PD-L1 inhibitor, durvalumab, in combination with a PARP inhibitor, olaparib, and a VEGFR1–3 inhibitor, cediranib, in recurrent women’s cancers with biomarker analyses
Source: J Immunother Cancer. 2019 Jul 25;7:197. doi: 10.1186/s40425-019-0680-3 (PMC6657373; doi:10.1186/s40425-019-0680-3)
Supplement: Supplementary file 1 — Figure S1. Pharmacokinetics effects of durvalumab on olaparib and cediranib. (A-B) Durvalumab did not affect olaparib PK Cmax or AUC. (C-D) The presence of durvalumab did not significantly affect cediranib PK. One patient’s PK data is missing due to no sample collection. One patient (red dot) showed abnormally low plasma concentrations that led to higher than normal CLss/F, possibly due to food effect on absorption. Abbreviations: AUCINF: area under the plasma concentration v. time curve from time zero to infinity. AUC/D: Area under the plasma concentration v. time curve normalized to dose. AUCTAU: AUC for the dosing interval for steady-state kinetics after durvalumab; 12 h for olaparib, 24 h for cediranib (PPTX 161 kb) [file 40425_2019_680_MOESM1_ESM.pptx]

## Slide 1
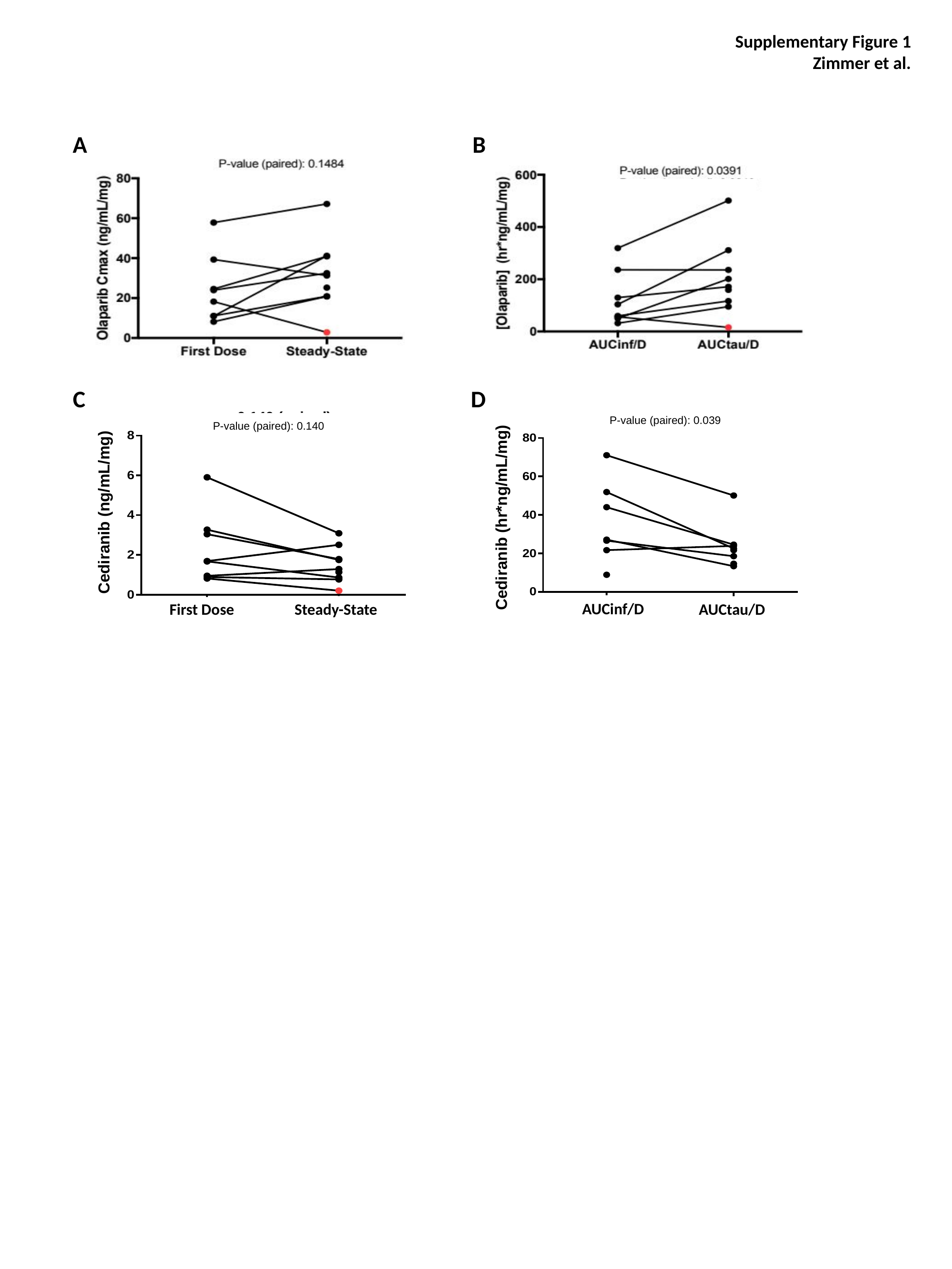

Supplementary Figure 1
Zimmer et al.
B
A
C
D
P-value (paired): 0.039
P-value (paired): 0.140
Cediranib (ng/mL/mg)
Cediranib (hr*ng/mL/mg)
AUCinf/D
Steady-State
AUCtau/D
First Dose
