# Supplementary figures and images for: A phase I study of the PD-L1 inhibitor, durvalumab, in combination with a PARP inhibitor, olaparib, and a VEGFR1–3 inhibitor, cediranib, in recurrent women’s cancers with biomarker analyses
Source: J Immunother Cancer. 2019 Jul 25;7:197. doi: 10.1186/s40425-019-0680-3 (PMC6657373; doi:10.1186/s40425-019-0680-3)

## Slide 1
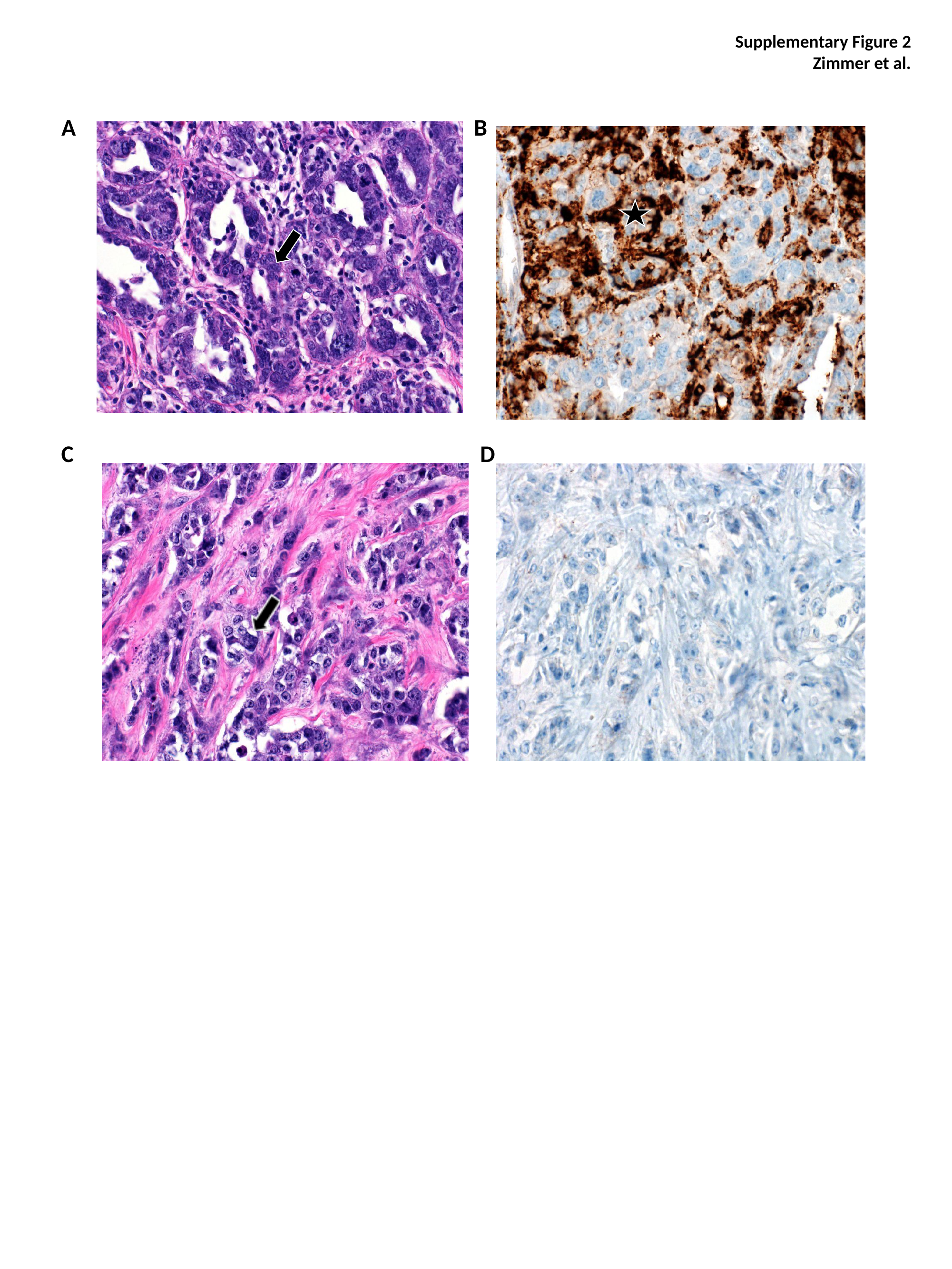

Supplementary Figure 2
Zimmer et al.
A
B
C
D

Supplement: Supplementary file 2 — Figure S2. Tumor infiltrating lymphocytes (TIL) and PD-L1 analysis by immunohistochemistry. (A-B) Patient B04 had a PR of 9 months duration; her primary HGSOC (arrow) showed PD-L1 positivity in the carcinoma cells, as well as within the TIL (star) (× 200). (C-D) Patient B09 experienced PD; her primary TNBC (arrow) did not display any PD-L1 labeling, and there were minimal TIL (< 5%) within the tumor bed. Abbreviations: PR: partial response, HGSOC: high grade serous ovarian carcinoma, TIL: tumor infiltrating lymphocytes, TNBC: triple negative breast cancer (PPTX 9168 kb) [file 40425_2019_680_MOESM2_ESM.pptx]

## Slide 1
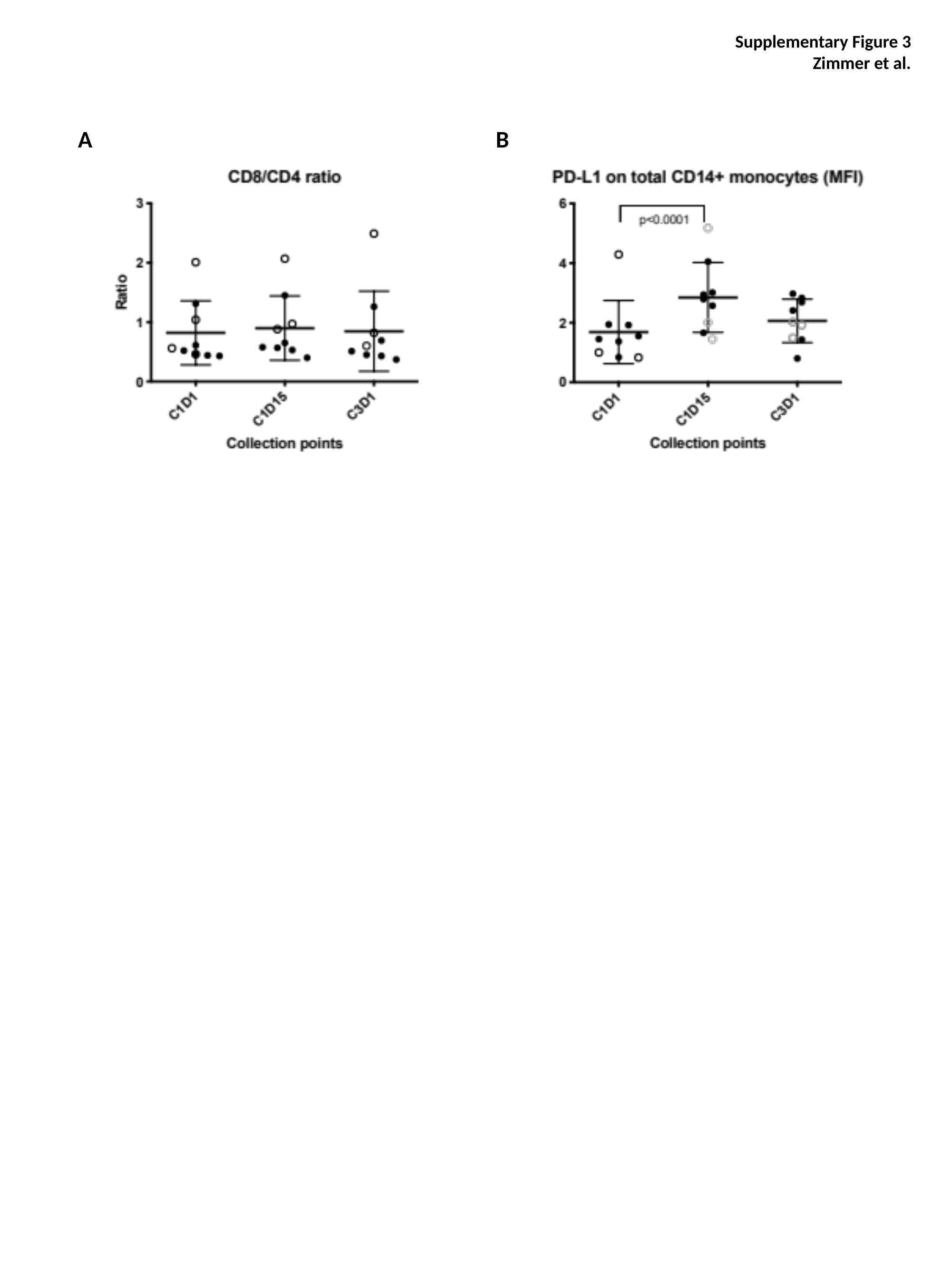

Supplementary Figure 3
Zimmer et al.
A
B

Supplement: Supplementary file 3 — Figure S3. Peripheral immune subsets and functional markers. (A) CD8/CD4 ratio. (B) PD-L1 expression on total C14+ monocytes. Open dots: germinative BRCA mutated cases. Abbreviations: MFI: median fluorescence intensity. (PPTX 95 kb) [file 40425_2019_680_MOESM3_ESM.pptx]

## Slide 1
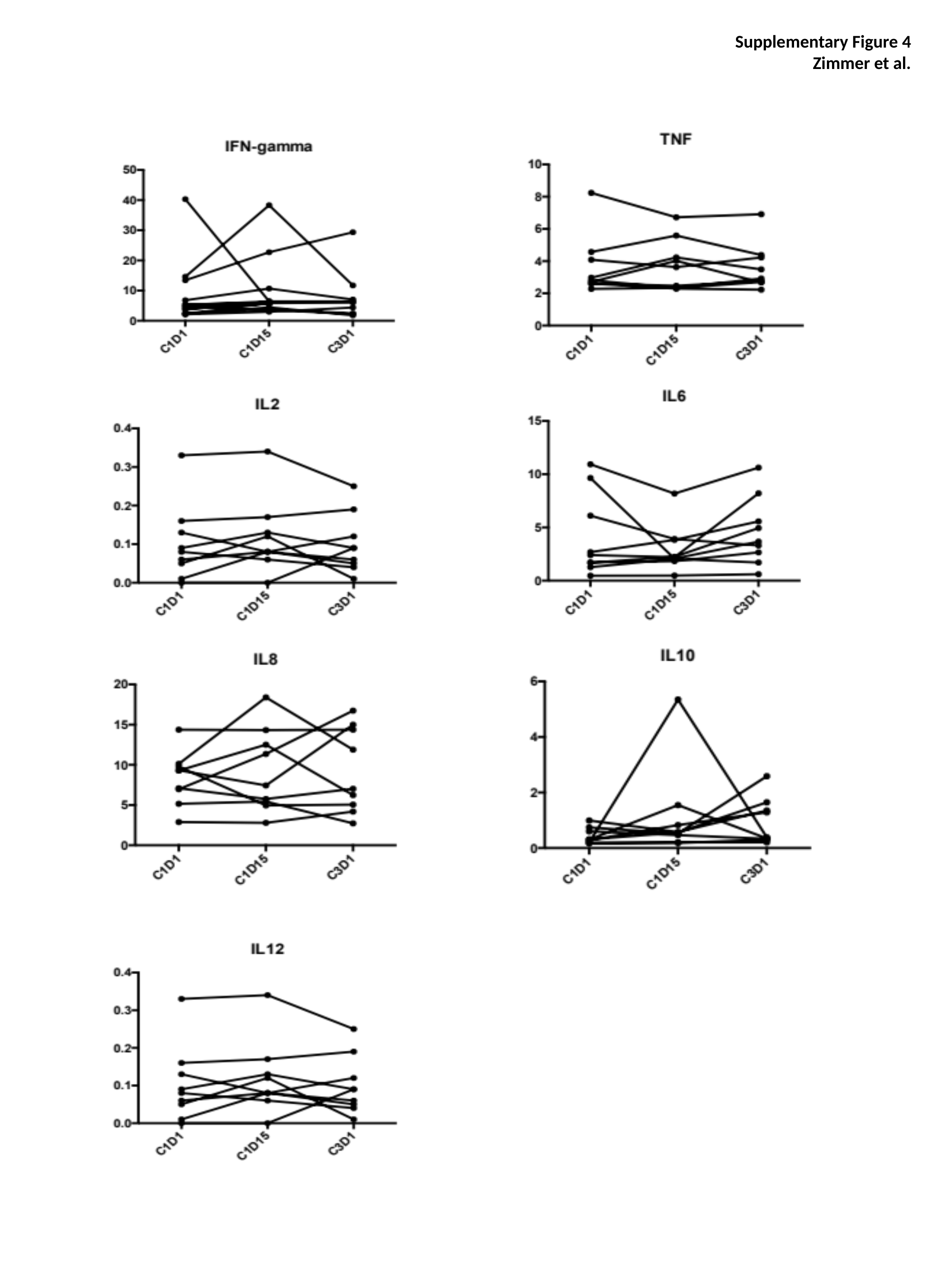

Supplementary Figure 4
Zimmer et al.

Supplement: Supplementary file 4 — Figure S4. Proinflammatory cytokines analysis. Plasma levels of pro-inflammatory cytokines (IFN γ, TNFα, IL 2, IL 6, IL 8 IL 10, and IL 12) were not changed significantly by the treatment. (PPTX 189 kb) [file 40425_2019_680_MOESM4_ESM.pptx]
