# Supplementary material for: A phase I study of the PD-L1 inhibitor, durvalumab, in combination with a PARP inhibitor, olaparib, and a VEGFR1–3 inhibitor, cediranib, in recurrent women’s cancers with biomarker analyses
Source: J Immunother Cancer. 2019 Jul 25;7:197. doi: 10.1186/s40425-019-0680-3 (PMC6657373; doi:10.1186/s40425-019-0680-3)
Supplement: Supplementary file 5 — Table S1. Pathologic characteristics and immune correlates. (DOCX 15 kb) [file 40425_2019_680_MOESM5_ESM.docx]

**Table S1. Pathologic characteristics and immune correlates**

|  | **All cases**  **(n=9)** |
| --- | --- |
|  | **Number (%)** |
| **Primary organ site** |  |
| Breast | 1 (11%) |
| Ovary/Fallopian tube | 7 (78%) |
| Uterus | 1 (11%) |
| **Primary tumor histology** |  |
| Triple negative breast carcinoma | 1 (11%) |
| Clear cell ovarian adenocarcinoma | 2 (22%) |
| High grade serous ovarian carcinoma | 3 (33%) |
| Malignant mixed mullerian tumor of the ovary | 1 (11%) |
| Mixed high grade serous and endometrioid ovarian carcinoma | 1 (11%) |
| Uterine endometrioid carcinoma | 1 (11%) |
| **Tumor tissue available for correlative studies** |  |
| Primary tumor | 8 (89%) |
| Metastasis at the time of diagnosis | 1 (11%) |
| **Treatment response** |  |
| Progressive disease (PD) | 2 (22%) |
| Stable disease (SD) | 3 (33%) |
| Partial response (PR) | 4 (44%) |
| **Carcinoma cell PD-L1 labeling** |  |
| Absent | 2 (22%) |
| >1% carcinoma cell labeling | 7 (78%) |
| **Tumor TIL infiltrate** |  |
| Mild infiltrate (score 1) | 3 (33%) |
| Moderate infiltrate (score 2) | 5 (56%)^†^ |
| Brisk infiltrate (score 3) | 1 (11%) |
| **Tumor TIL PD-L1 labeling** |  |
| Absent (score 0) | 1 (11%) |
| Focal PD-L1+ TIL (score 1) | 3 (33%) |
| Moderate PD-L1+ TIL (score 2) | 3 (33%) |
| Brisk PD-L1+ TIL (score3) | 2 (22%) |

Abbreviations: n:number; TIL:tumor infiltrating lymphocytes

^†^Two patient’s tumors that contained moderate TIL also displayed tertiary lymphoid structures/lymphoid aggregates; one of these sites was a metastasis to the pericolonic soft tissue at the time of diagnosis
